# Case of Death from Fractured Femur

**Published:** 1850-10

**Authors:** S. B. Mills

**Affiliations:** Resident Physician, City Hospital, San Francisco, California


*Case of death from fractured Femur.* Reported by S. B. Mills, M. D., Resident Physician, City Hospital, San Francisco, California.

William Toppin, entered the city hospital of San Francisco, Feb. 29th, with fracture of the right femur, having received said injury in a fit of intoxication, by falling down a precipice at "Clark's point," and having been treated on board the U. S. ship of war Warren,—— Assist. Surg., for some months previous. At a consultation of the most eminent men of this place, and at the earnest solicitation of the patient, it was concluded prudent to operate, and consequently Dr. Peter Smith, city physician, operated, Jan. 19th, for false joint. The patient's bad condition of health made it impossible to hope for much good, but in a desperate case we employed a desperate remedy. The said William Toppin died June 20th.

On examination we found a transverse fracture with the ends of the bones dove-tailed; the ends of the bones remaining healthy with a very slight inflammation of the periosteum.

[The above case we deem of great importance to the profession. It shows how careful a surgeon should be in ascertaining the exact position of the fragments of fractured bones; and in their coaptation. In this case we understand, the former were widely separated by an interposing muscle; and if such were the fact, would it not have been better, even at the hazard of making a compound fracture, for the surgeon to cut down and remove the muscle from between the fragments, after all other resources had failed, rather than to wait, as was done, until so much callus had been thrown out; and the swelling of the thigh had so increased, that the condition of the fragments could not be ascertained? G. R. B. H.]

